# A scoping review of post-earthquake burnout of doctors/nurses and gaps in preventive and GIS-supported interventions: case of Türkiye and Syria

**DOI:** 10.1186/s12889-026-27459-1

**Published:** 2026-04-24

**Authors:** Filiz Ruhm, Elif Oksan Çalıkoğlu, Mehmet Karadağ, Beril Bayrak Bulucu, Baran Çalışgan, Zaina Chaban, Habib Salamah, Sohrab Safi, Seyedeh Ala Mokhtabad Amrei, Uğur Berke Kurt, Patrick Marius Koga

**Affiliations:** 1 Dept. of Political Science, Plymouth State University, Plymouth, NH USA; 2 Dept. of Public Health, School of Medicine, Gaziantep University, Gaziantep, Türkiye; 3 Dept. of Child and Adolescent Psychiatry, School of Medicine, Gaziantep University, Gaziantep, Türkiye; 4 Brazelton Institute, Boston Children’s Hospital, Boston, MA USA; 5 Zeru Foundation, 501(c)(3) nonprofit, Westford, MA USA; 6 Ulysses Refugee Research Program, PHS Dept., School of Medicine, University of California, Davis, CA 95616 USA; 7 School of Medicine, University of California, Davis, USA; 8 University of Health Science Gülhane School of Medicine, Ankara, Türkiye; 9 Department of Public Health Sciences, School of Medicine, University of California, Davis, CA USA

**Keywords:** Post-earthquake burnout, Doctors, Nurses, Preventive and early intervention models, GIS, Türkiye

## Abstract

**Background:**

Disasters like earthquakes severely affect healthcare systems and personnel. While outcomes of psychological burdens, including burnout, secondary traumatic stress, and emotional exhaustion of healthcare workers are well documented, less research is known about the availability and effectiveness of structured preventive and early intervention (PEI) strategies, particularly those supported by system-level or data-driven approaches, like Geographic Information Systems (GIS).

**Methods:**

Based on a systematic search of major data bases, this study reviews 35 years of literature (1990–2024) on post-earthquake burnout among doctors and nurses in Türkiye and Syria, with a focus on identifying the extent to which PEI models—especially those incorporating Geographic Information Systems (GIS) or similar tools—have been developed or evaluated. Eligible studies were screened and analyzed using an inductive thematic approach.

**Results:**

Our review confirmed elevated burnout and psychological distress among healthcare workers following earthquakes (natural disasters), with nurses often experiencing disproportionately higher levels of emotional exhaustion. However, the literature also revealed a limited presence of structured PEI models and an almost complete absence of GIS-supported or system-level intervention frameworks.

**Conclusion:**

Our findings show a critical gap between the documented burden of post-disaster burnout and the limited development of structured intervention models. The near absence of GIS-informed or system-level approaches in the reviewed literature suggests a critical gap and an important direction/opportunity for future research and public health practice, particularly in disaster-prone regions.

## Introduction

The Türkiye-Syria earthquakes of February 6, 2023, were the largest in the region since 1939. More than 72,000 people have died, 160,000 have been injured, and over 1.5 million were left homeless in Türkiye [1]. On February 10, 2023, Türkiye's Health Minister announced that 18,022 doctors and 111, 699 health personnel were working at the quake sites as well as 14, 435 National Medical Rescue Team members [2]. Additionally, more than 13,000 emergency personnel and volunteers from 100 nations were dispatched to the earthquake zones [3, 4]. On February 13, in the first post-earthquake week, the Turkish Medical Association said that 67 doctors based in Adana, Diyarbakır, Adıyaman, Hatay, Kahramanmaraş, Malatya, and Osmaniye, died in the earthquakes [2].

Witnessing the catastrophic destruction caused by a disaster can be overwhelmingly traumatic [5]. While the mental impacts on survivors have been abundantly reported, the psychological trauma and the professional burnout of doctors and nurses working in the disaster response is often overlooked [6]. Moreover, some healthcare professionals are themselves earthquake survivors worrying for their own families, coping with personal losses and anxieties while continuing to fulfill their duties. Acknowledging their dual, provider and survivor, role is essential for shaping interventions that support their resilience. The physical and psychological demands of rescue work often result in exhaustion, compassion fatigue, and reduced resilience [6]. Doctors and nurses are faced with a heavy moral burden as they must make heartbreaking decisions about patient care prioritization. As Nishi et al. (2012) and Pathwary et al. (2023) note, they may become overwhelmed with grief, guilt, perceived professional failure, and may succumb to acute or peritraumatic stress disorder. A high risk for severe mental distress is ever present for rescue workers [6]. The Ozen, S., & Sir, A. (2004) study has found that a quarter of the personnel serving in the 2003 Bingol earthquake in Türkiye developed PTSD.

Working in the aftermath of an earthquake, doctors and nurses find themselves pressed to serve beyond their scope of training and to work in unsafe buildings, with dead bodies, gruesomely injured, highly distressed, or aggressive survivors; as such they are at an elevated risk of developing PTSD. Lacking disaster training and skills, professional and non-professional volunteers may develop PTSD especially when they are uncertain about their responsibilities. A challenge is the lack of resources tailored to support the mental health needs of rescue workers. The Patwary study (2023) aptly notes that as paramount priority in a disaster is ensuring essential medical care, food, water, blankets, and shelter for survivors, mental health support for frontline personnel become a secondary concern or is altogether neglected.

The devastating aftermath of the 2023 earthquakes in Türkiye and Syria was also compounded by a severely limited mental care. The significant shortage of psychiatrists in Türkiye, 6.63 psychiatrists per 100,000 people, the lowest ratio in Europe [7], is aggravated by geographic disparities: the majority of psychiatrists practice in large cities and the western parts of the country while Eastern Anatolian rural areas are left with long wait times to access mental support. Last, but not least, social stigma can be an added barrier. Many rescue workers avoid seeking support fearing social stigmatization as mentally vulnerable or professionally inadequate [8]

While the psychological impacts of disasters on healthcare professionals have been extensively documented, extremely limited attention has been paid to the development of structured, system-level PEI frameworks. Existing research tends to focus on individual-level outcomes like burnout, secondary traumatic stress, and emotional exhaustion. These studies do not address how these risks might be anticipated, monitored, or mitigated, in particular, through coordinated, data-driven and geospatial tools such as GIS, which offers potential for mapping workforce strain, identifying high-risk regions, and supporting targeted interventions. This review therefore seeks to assess not only the documented burden of burnout but also the presence—or absence—of structured PEI models, including those supported by GIS or similar system-level tools.

Due to the various published manuscripts and the findings as related to this scoping review, the terminology used in this manuscript consciously presents the text as noted in these publications found. Terms of “review, burnout, emotional exhaustion, secondary traumatic stress, and compassion fatigue” are therefore treated as related but distinct constructs.

### Nurses burnout

It must be emphasized that burnout is not an individual mental health diagnosis [9]. Coined in the 1970 s by the American psychologist Herbert Freudenberger, the term "burnout" was added only in 2019 to the International Disease Classification—ICD 11 as an “occupational phenomenon and not a medical condition” [9]. Within WHO's taxonomy bureaucracy, formalizing this “phenomenon” took almost 50 years. In the chaotic circumstances of disasters, nurses triage and prioritize patients to allocate effectively limited resources [10]. While some nurses can provide psychological first aid and counseling support to survivors, many do not have this expertise. [11, 12] A 2020 study of nurse preparedness in disaster management in Indonesia, pointed out that while 54.33% of 147 nurses were able to coordinate efforts with other professionals, 45.77% of them were not trained enough to do so [11, 13]

Nurses appear to be especially vulnerable to the extreme stress of disasters impacting them at personal, family-related, professional, and social levels [14]. The findings of Vagni et al. (2020) show that such spiking vulnerabilities leave many nurses physically and emotionally exhausted, numb, irritable, struggling with insomnia, intrusive thoughts, and a reduced work performance. Proactive measures like disaster training, clear communication protocols, mental health support, can significantly improve resilience, effectiveness, and overall the disaster response performance [12]. Although many studies report negative impacts of disasters on nursing performance, there is still a lack of a comprehensive understanding of this issue. Some studies focused on the nurses' roles in disasters, other studies only assessed their disaster knowledge, attitudes and competencies without looking at personal vulnerabilities [15]. Scoping and systematic reviews could provide better filtered information on disaster burnout. A comprehensive understanding of nurses' experiences during disasters could improve their preparedness through disaster training and continuing post-disaster support [16].

### Medical doctors burnout

Physician burnout affects not only the doctors but also their patients. The findings of a 2022 study on physicians working in Caribbean emergency departments, using the Maslach Burnout Inventory (MBI) and the Resilience Scale-14 (RS14) as measures of burnout and resilience suggested that physicians were 15 times more likely than other professionals to suffer from burnout, high rates of coronary artery disease, peptic ulcer disease, drug abuse, and sleep disturbances [17]. While mental effects of burnout in doctors include emotional exhaustion, depersonalization, cynicism [18], PTSD and suicidal ideation [19], risks also extend to patients because of higher rates of medical errors and adverse clinical outcomes [17, 20]. Furthermore, emergency medicine doctors have higher burnout rates than colleagues in other specialties [21]. While many studies have investigated multiple factors involved in physician resilience and burnout, we still lack a coherent understanding of how these determinants interact to shape an outcome [22]. Most physician burnout research has focused on its more proximal determinants of physician burnout [23], linking gender [24], career stage [25], work stress [26], organizational inefficiency [27], and workplace culture [28]. Less attention has been given to distal determinants such as system-level and inter-system pressure. During disasters, inadequate preparedness, local disparities, poverty, clinic- or health system-level resource shortages, and conflicting communication, interact to escalate stress [29]. This raises the question of how structural realities intersect during disasters to shape physician burnout [30] especially when the speed and complexity of these intersections surge exponentially [31]. Furthermore, this points out that while individual training and support are important, medical burnout, as a primarily workplace phenomenon, calls for organizational preparedness and large-system proactivity and solutions. And yet, even the best systemic readiness may not be foolproof in the unpredictable and time compression reality of a disaster [32].

### Pressing need for fast information flow in disaster management

Pressure for fast information in time of disaster can easily overwhelm the capacity of individuals and systems [33]. Surging uncertainty and escalating threats press health systems to provide immediate, accurate information [34]. Proactively monitoring doctors' and nurses' vulnerability and resilience to a potential disaster burnout is an imperative for decision makers [35].To plan an informed response to earthquakes, public health systems need analytic methods to quickly estimate the number of people who will be affected, the subpopulations at particular risks, and to locate and quantify hospitals, schools, and other facilities needed during a response [36]. Given the complexity and the lengthy lead time required for local health officials to prepare personnel, facilities, and medical supplies for a public health response, establishing a baseline dataset in advance of a disaster is vital [37]. Various burnout vulnerability and resilience indexes can be computed and mapped using streaming live standardized data feeds into GIS. In turn the GIS could offer rich geospatial analysis functions for network infrastructure systems that involve geographic references. Linking these data to geospatial modeling could automatically calculate in real time vulnerability and resilience indexes for doctors and nurses thus supporting intersectoral, interprofessional work [38].

To scope out the post-earthquake burnout of doctors and nurses and the uptake of GIS in prevention and early intervention models in Türkiye we conducted a review of the past three decades of literature. As a form of “knowledge synthesis” [39], a scoping review explores and filters existent literature to inform researchers, practitioners, and policy makers on the magnitude of a knowledge base and its possible gaps. The 1990–2024 timeframe was selected to capture both pre-digital and contemporary disaster response literature, allowing assessment of how technological approaches, including GIS, have (or have not) been integrated over time. Through this scoping review, we aim to discuss and respond to the question: What does the literature reveal about (1) the nature of post-earthquake burnout among doctors and nurses, and (2) the extent to which PEI models, including GIS-supported or other system-level approaches, have been developed, implemented or evaluated?”.

## Methods

This review aimed to probe the 1990–2024 peer-reviewed literature on post-earthquake PEI models for doctors and nurses’ burnout in Türkiye and Syria. The methodological approach for our review was grounded in the [40] five step framework enhanced by extensions and recommendations by [41, 42]. In our experience, this enhanced method "integrates qualitative and quantitative literature assessments and employs an interprofessional, team-based approach through all stages of the scoping review" [43]. We used the Preferred Reporting Items for Systematic Reviews and Meta-Analyses (PRISMA), the Extension for Scoping Review guidelines, and the PRISMA-ScR Checklist and Guidelines [44].

### Step 1: identifying the research question

First, we conceptualized a review question informed by several exploratory searches. Further, we used backward and forward searching to optimize your queries This quick scoping suggested that research on post-disaster burnout among doctors and nurses spans a wide array of fields and disciplines. This understanding encouraged us to integrate diverse professional perspectives into a collaborative review framework. Consequently, and in alignment with Westphal’s enhanced scoping review model, we recruited additional reviewers to widen the span of our expert skills, perspectives, and professional experiences. Our transdisciplinary group included a postdoctoral research fellow (S.S.), three students (U.B.K.-medicine; H.S.-medicine; SAM-premed), and six faculty (E.O.C.; M.K; B.C.; F.R.; B.B.B.; P.M.K.) from five schools of medicine, public health, and political science with extensive expertise in global health, epidemiology, refugee health, psychiatry, governance and human rights, medical anthropology, and health informatics. The team was trained in scoping and systematic reviews, PRISMA, and Covidence, a systematic review software [45]. For six months, authors from different disciplines met regularly to examine and discuss the data. The benefit of such meetings is that it avoids monodisciplinary bias [46]. Additionally, they support the emergence of a integrative approach to interprofessional perspective exploration, data analysis, and clarification of themes and subthemes [47, 48].

### Step 2: identifying relevant studies

Scopus, Embase, PubMed, and PsychInfo databases were mined for studies published during January 1990 and December 2024. The search strategy used the following terms: post-earthquake burnout/medical doctors/nurses/post-disaster/preventive interventions/Geographic Information Systems/disaster planning/Türkiye. Searches were conducted between May – December 2024. 147 article titles and abstracts were imported into Covidence for screening.

### Step 3: study selection

#### Inclusion and exclusion criteria

**Fig. 1:**
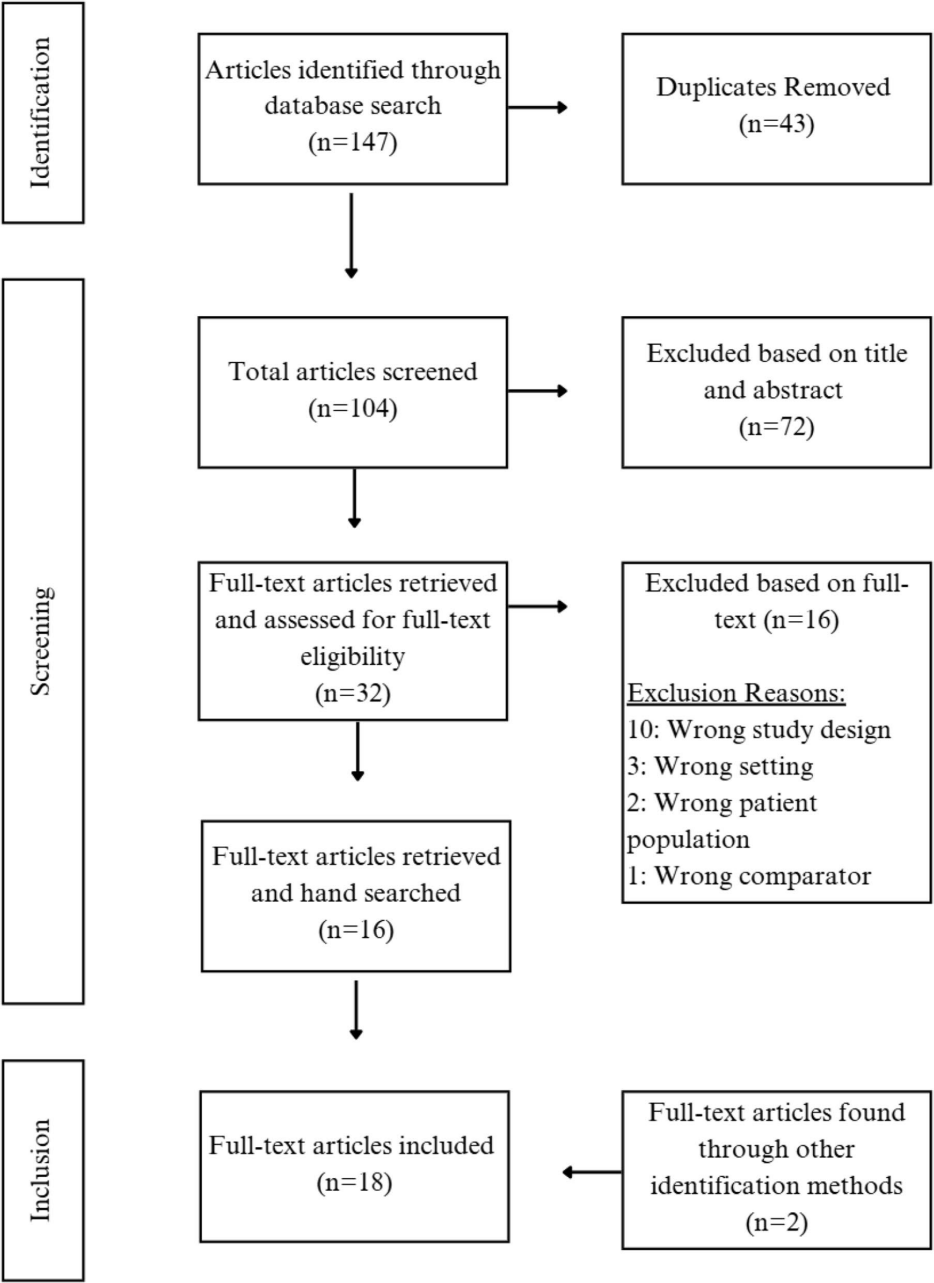
PRISMA flow diagram

After identifying relevant literature, we assessed the articles using the inclusion and exclusion criteria (Table 1). The PRISMA flow diagram (see Fig. 1) reported our search yield and article selection process. Although inclusion criteria allowed for PEI-related studies, most included articles did not explicitly define or evaluate structured PEI models.

**Table 1:** Inclusion and exclusion criteria

Inclusion	Exclusion
1. Peer-reviewed papers 2. Doctoral dissertations 3. Published between 1990–2024 4. Published in English or in Turkish 5. Qualitative and quantitative research, scoping, and systematic reviews 6. Studies on post-earthquake disaster in Türkiye 7. Studies included Turkish doctors and nurses 8. Earthquake PEI models mentioned explicitly or implicitly 9. Studies addressed burnout vulnerability and/or resilience	1. Grey literature (e.g., reports, policy papers, statistical data) 2. Editorials, book chapters, commentaries 3. Expert opinion, cohort reports; RCTs 4. Studies outside of Türkiye or Syria 5. Have not included Turkish doctors and nurses 6. Earthquake PEI models not mentioned or implied 7. Burnout vulnerability and/or resilience have not been addressed

Duplicates were automatically removed from Covidence. Four researchers (SS, SN, HS, and SAM) screened in tandem by title and abstract the remaining 104 articles. Disagreements were resolved by an arbiter (BC). After excluding 68 records, only 32 articles were selected for a full-text analysis. Four researchers (EOK, MK, FR, and BBB) reviewed full texts in tandem with the senior author (PMK) as arbiter; all conflicts were resolved through consensus. We assigned a unique identifying number to each article to be included in the full-text review. We filtered 32 records down to 16 articles that met our eligibility criteria. To these, two more articles identified in Turkish databases were added (see Table 2).

**Table 2:** Charting of the finding (next page)

	**COLUMN 1** **Author information**	**COLUMN 2** **Study Design & Participants**	**COLUMN 3** **Research Aims**	**COLUMN 4** **Burnout of doctors and nurses serving as disaster relief workers**	**COLUMN 5** **Vulnerability of doctors and nurses as survivors living in affected communities**	**COLUMN 6** **PEI Model used, evaluated or suggested** **(ecological; GIS; mHealth; etc.)**	**COLUMN 7** **Research, practice, and/or policy recommendations**
1	Özbay, A., & Bülbül, A. E. (2025). The impact of psychological resilience and gender on the relationship between trauma-coping perception and levels of secondary traumatic stress in mental health workers. Journal of community psychology, 53(1), e23150. https://doi.org/10.1002/jcop.23150	Qualitative survey of 212 mental health workers from various provinces of Turkey to support the 2023 earthquake survivors	Study examined the mediating role of psychological resilience and gender on mental health workers' perceptions of coping with trauma and secondary traumatic stress	Burnout not explicitly assessed	The vulnerability of mental health workers was assessed using the “Perception of Coping with Trauma Scale,” “Secondary Traumatic Stress Scale,” “Brief Psychological Resilience Scale. Male HCWs appear to be more vulnerable than females	No PEI model has been explicitly identified	Create gender‐specific intervention and support programs, particularly for men, to prevent the adverse effects of secondary traumatic stress. Periodic psych. counseling and therapy services should be available to mental health professionals
2	Balikuddembe, J. K., Reinhardt, J. D., Vahid, G., & Di, B. (2024). A scoping review of post-earthquake healthcare for vulnerable groups of the 2023 Turkey-Syria earthquakes. BMC public health, 24(1), 945. https://doi.org/10.1186/s12889-024-18395-z	A scoping review (Arksey and O’Malley framework). PubMed, Science Direct, Web of Science, OVID, and Google Scholar searched for studies published between March and April 2023	This study aimed at identifying the interventions undertaken or proposed for addressing the health needs or challenges of vulnerable groups immediately after the occurrence of the 2023 Turkey- Syria earthquakes, as well as for prioritizing their healthcare service delivery in the post-Turkey-Syria earthquake	Burnout not explicitly assessed	The most vulnerable and affected groups by the 2023 Kahramanmaraş earthquakes also included rescue, medical, and HCWs. These groups were disproportionately affected and overlooked in prehospital and hospital-based emergency preparedness and response plans. HCWs physically and emotionally overwhelmed and at high risk for anxiety and trauma	There were no streamlined or context-specific recs for addressing post-earthquake healthcare services (PEHS) for vulnerable groups. Most studies proposed the mental health and psychosocial support (MHPSS) to target the affected victims with post-mental health and psychosocial symptoms and conditions such as post-traumatic stress disorders, stigma, anxiety, and depression	MHPSS proposed services included telepsychology, training mental HCWs, self-care resources, creative and group-based mental therapeutic sessions, and community mental outreach. MHPSS services to be integrated into the primary healthcare systems of Turkey and Syria and be augmented by mobile health clinics to provide specialized mental healthcare to the most vulnerable groups
3	Sani Mert, I., & Koksal, K. (2025). Unveiling the heart of disaster nursing: A qualitative study on motivations, challenges, and lessons from the devastating 2023 Turkey earthquakes. Intl. nursing review, 10.1111/inr.13023. https://doi.org/10.1111/inr.13023	A descriptive qualitative study conducted between March- May 2023 using semi structured interviews with 15 nurses who volunteered to work in the earthquake zone	This study analyzed nurses’ experiences before, during, and after deployment in response to the 2023 Turkey earthquakes to enhance disaster-response efforts	Used the Turkish version of the Nurse Stress Scale (NSS). Stress factors: Death and dying; Inadequate prep. to deal with the emotional needs of patients and their families; Conflict with physicians, with other nurses and supervisors; Lack of staff support, uncertainty about treatments	Insufficient training and coping with anxiety and stress were common themes	No PEI model has been explicitly identified	Train nurses in disaster preparedness. Offer drills, simulations, online courses, workshops, and collaborative partnerships. Provide psych. support for emotional challenges. Regularly update response policies based on past experiences/
4	Düzgün, G., Polat, G., Hakverdioğlu Yönt, G., Şenuzun Aykar, F. (2023). Izmir Earthquake Experience of Healthcare Professionals During Pandemics: A Qualitative Study. Health Academy Kastamonu, 8(2), 227–236. https://doi.org/10.25279/sak.1141607	A qualitative study (phenomenological approach) to investigate the experiences of 11 participants (incl. 6 nurses), with the earthquake. Semi-structured interviews were used to describe experiences, meanings, and personal impacts	This study aimed to assess the impact of emotions on occupational competence and performance of healthcare professionals in providing care to earthquake victims. The study did not use any performance or burnout assessment scales	Inadequate training to face the 2020 Izmir earthquake. Most nurses experienced fear, shock, panic, treating survivors, scarce resources. Logistical chaos, assignment ambiguity, insufficient coordination in disaster response, high workload, and burnout	Most nurses are very anxious about their and their families’ health and this led to their emotional exhaustion	No specific model identified. Nurses demonstrated good skills in communication, problem-solving, empathy, patience, tolerance, and understanding. This enabled them to overcome difficulties and work seamlessly together in spite of logistical challenges	Enhance training to improve triage, decision-making, teamwork, adaptation and psychological preparedness. Prepare to role-play disaster scenarios, drill practices, evaluate mental and professional performance and update hospital disaster plan
5	Deniz Doğan, S., Köse Tosunöz, İ., Kaya, P., Yurtseven, Ş., Aydinli, A. (2024). Being a nurse in Turkey's disaster: A phenomenological study on post-earthquake experiences International Journal of Disaster Risk Reduction 02/01 2024;103():104,346 https://doi.org/10.1016/j.ijdrr.2024.104346	A qualitative study of 30 nurses who provided care in Adana and Şanlıurfa) in 2023 to survivors of the Kahramanmaraş̧, earthquakes	Using a phenomenological approach, this study aimed to investigate the lived personal experience and the work challenges of the participants	Inadequate organization, shortage of resources and personnel to handle a heavy workload beyond the hospital's capacity. Insufficient knowledge about disaster patient care, difficulty adapting. Poor coordination, lack of unity in command, and inadequate management. Unsafe hospital buildings with large cracks. Fear had a negative impact on nursing care and produced burnout	Nurses afraid of aftershocks, worried about their families. Loneliness, tiredness, and helplessness in addition to the emotional burden experienced by nurses as earthquake survivors themselves. At the same time, nurses had to be the distressed patient's primary source of support. All t nurses felt inadequate, vulnerable, and burned out	No PEI model has been explicitly identified	Nurses have gaps that need to be filled with disaster preparedness and basic competencies. Nurses caring for earthquake victims need various training and support mechanisms both in patient care and in self-care
6	Salik, H., Şahin, M., & Uslu, Ö. (2024). Experiences of Nurses Providing Care to Individuals in Earthquake-Affected Areas of Eastern Turkey: A Phenomenological Study. Journal of community health nursing, 41(2), 110–122. https://doi.org/10.1080/07370016.2023.2285964	A qualitative study conducted between May 29, 2023, and August 15, 2023, with 11 nurses working in the eastern Anatolia's provinces through one-on-one semi-structured interviews	The study aimed to examine the experiences of nurses who provide care to individuals affected by the Kahramanmaraş̧ earthquakes	Insufficient knowledge and crisis management skills to care for victims. Difficult environment (hygiene, cold weather, shelter) and work-related (lack of coordination, psychosocial issues) conditions	Feelings of sadness, inadequacy, anxiety, and fear	No PEI model has been explicitly identified	It is recommended that nurses are provided with realistic training through simulations and drills in disaster management, as well as psychological support interventions
7	Emirza, E. G., Uzun, S., & Şenses, M. (2024). Earthquake diaries: Psychosocial difficulties and life experiences of nurses working in the disaster zone after the earthquake: A phenomenological study. Public health nursing (Boston, Mass.), 41(5), 1124–1134. https://doi.org/10.1111/phn.13369	Qualitative study, semi-structured in-depth interviews with 18 nurses who provided direct health care services to earthquake victims	This study aimed to evaluate the psychosocial difficulties and life experiences of nurses working in the disaster area following the Februaryruary 6, 2023 Kahramanmaraş earthquakes	Worrying/sadness for people, difficulty in providing care services while witnessing intense destruction and working with limited resources. Unsafe buildings, risk of endemic infectious disease transmission, long working hours, crying, staff shortages, physical fatigue, anxiety, burnout, and PTSD	The Februaryruary 6, 2023 earthquake caused secondary traumatization in nurses, they had great difficulty in coping with their own traumatic stress. The personal vulnerability of many nurses increased their professional work burnout	No PEI model has been explicitly identified	It is recommended to organize psychosocial support and PTSD rehabilitation programs for nurses to strengthen resources such as social support, effective coping strategies and to increase solidarity
8	Akçay et al. (2025). Emotional exhaustion and psychological distress among health care workers after the 2023 Kahramanmaraş earthquake: Prevalence and associated factors. The American journal of orthopsychiatry, 10.1037/ort0000792. Advance online publication. https://doi.org/10.1037/ort0000792	An observational cross-sectional study conducted on a sample of 71 doctors and 92 nurses working in Ankara children’s hospital where earthquake victims were removed first referred and treated. None of the health workers were directly exposed to the earthquake	The study aimed to compare psychological distress, burnout, and resilience of different health professionals in the early post disaster period. A second aim was to explore the factors affecting high burnout in the most vulnerable health care worker group emotionally exhausted	Burnout, psychological distress, and resilience assessed with Maslach Burnout Inventory, the Depression and Anxiety Stress Scale–21, and the Brief Resilience Scale. 54.9% of participants reported high emotional exhaustion; half of those were nurses. Nurses had higher emotional exhaustion, depression, and stress scores as well as low self-reported resilience	The high stress of early post-disaster period (shiftwork; high workload; constant exposure to children’s suffering and death, etc.) burdens doctors and nurses, affecting their mental health and resilience. The lower resilience in nurses suggests that their high vulnerability to negative emotional states has led to higher emotional exhaustion and burnout than in doctors	No explicit ecological and/or GIS PEI model has been identified	Health care organizations should support frontline nurses, particularly those with less work experience, by making resources available to address stressors and with training to improve their resilience during and post disasters
9	Yanik, D., & Ediz, Ç. (2024). Experiences and psychosocial challenges of volunteer nurses in Turkey devastating earthquake zones: Lessons to be learnt for prevention of health system problems in disasters: A qualitative study. Public health nursing (Boston, Mass.), 41(3), 503–513. https://doi.org/10.1111/phn.13303	A qualitative study employing semi-structured, in-depth interviews with 15 volunteer nurses who were actively working in 2023 in earthquake -affected provinces of Hatay, Gaziantep, Adıyaman, Kahramanmaraş, and Malatya	This research aimed to investigate experiences and psychosocial challenges encountered by volunteer nurses who provided care in the zones affected by the devastating earthquake that struck Turkey in 2023	Lack of disaster training and of translators. Long wk. hours, fatigue. Disparities in practices among foreign personnel Management failures: housing, food, drinking water, toilet, bathroom and hygiene, heating, clothing, security, insuff. materials, meds, and medical equipment. Admin. failure to establish crisis coordin., to obtain interv. permits, to allocate personnel. Incompetent, and inexperienced managers	Severe psychosocial problems: mourning; stress; fear; sorrow; desperation; guilt; anger; depression; crying; insomnia, nightmares; flashbacks; ineffective coping (smoking) and suppression No privacy	No explicit ecological and/or GIS PEI model has been identified However, an implicit, unnamed model has been evaluated qualitatively by the nurses	Weak recommendations; merely to plan ahead for training in disaster nursing
10	Özbek Güven, G., Karataş, M., & Kaynak, S. (2024). Trauma levels and perspectives on dignified death among nurses and physicians who directly experienced the recent earthquake. PloS one, 19(10), e0311184. https://doi.org/10.1371/journal.pone.0311184	A cross-sectional study conducted between October 1 and December 31, 2023, using with 280 nurses and 280 doctors working in hospitals in Kahramanmaraş̧, Hatay, Malatya, and Adıyaman hospitals providing care to victims	To determine the levels of trauma experienced by physicians and nurses who experienced the earthquake, their perceptions of a "good death", and to understand the relationship between these factors	Disaster-affected health care workers (doctors and nurses) fatigued by long working hours. Burnout not directly assessed	Compared to physicians, nurses were more sensitive to trauma and "psychosocial spirituality and good death". Nurses were emotionally and psychologically more affected by the death of their patients, were more contact with patients and their relatives compared to physicians	No explicit ecological and/or GIS PEI model has been identified	Evaluate the perceptions of a good death among professionals to strengthen their coping and communication with patients. Provide disaster support, resources, and trainings, especially for nurses, Develop policies to support their emotional and psychological needs to enhance their resilience
11	Satılmış, D., Yıldız, E., & Cevik, E. (2024). Posttraumatic stress disorder in health-care workers after two major earthquakes centered in Kahramanmaraş, Turkey. Turkish journal of emergency medicine, 24(1), 27–32. https://doi.org/10.4103/tjem.tjem_192_23	A multicenter study conducted 2 months after the 2023 Turkey earthquake among 79 HCWs, including doctors and nurses working in the region	Trauma signs are common in HCWs. If these symptoms are not diagnosed and treated promptly, they can turn into PTSD, resulting in poor performance, absenteeism, and burnout. This study aimed to investigate the risk factors for PTSD and the factors in HCWs by using the PTSD Checklist for DSM‐5 (PCL‐5) scale	HCWs who do not have disaster training or who have not previously encountered similar cases experience psych. distress when dealing with the suffering of patients and families, with long working hours, insomnia, and life‐threatening situations	PTSD rates were high among HCWs with female HCWs at higher risk than males. The female gender was a risk factor for PTSD because nurses were in closer contact with the victims after an emergency, have experienced more identification with their patients, and may have felt guilty when faced with a failed outcome	Turkey’s Disaster Response Plan (Türkiye Afet Müdahale Planı—TAMP), was activated at level four in response to the earthquake crisis, and all disaster response teams were mobilized promptly. However, no explicit ecological and/or GIS PEI model has been identified	HCWs, especially females working in the disaster area, should be closely monitored, and more mental health services should be provided to ensure that HCWs receive the necessary support in the post disaster period
12	Sayin D, Kubuk B, Bayraktar E, Cariou M, Gömleksiz İ, Sürmeli A. (2024). Deploying a user-friendly GIS mapping tool in post-earthquake Turkey and Syria. J. glob. health econ. policy. 2024;4:e2024006. https://doi.org/10.52872/001c.125043	A performance eval. study of an interactive GIS tool, coupled with an integrated WhatsApp chatbot, intended to redress the lack of real-time, accessible data on 537 healthcare facilities in the disaster areas	The study aimed to evaluate the implementation of digital health tools for enhancing healthcare delivery and coordination in the aftermath of the 2023 Turkey-Syria earthquakes The GIS disaster health map included the display of data from 537 health service centers	Burnout not assessed. However, real-time solutions in post-crisis settings play a major role in coordination between stakeholders involved in the response and can reduce responders’ burnout	Personal vulnerability not assessed in this study	Used HERA**,** a GIS tool, https://afetharitasi.org/ and a WhatsApp- based Chatbot to facilitate real-time data collection, communic. and visualization among healthcare providers and responders. The digital health tool has been effective in enhancing disaster response	Use HERA tool to solve coordination issues in post-earthquake low resources settings (disruption of electricity and access to internet) Recommendations include integrating user-centered, deployable technologies to improve healthcare delivery in emergency earthquake settings
13	Çifçi, S., & Kilinç, Z. (2024). The Disaster of the Century: Effects of the 6 Februaryruary 2023 Kahramanmaraş Earthquakes on the Sleep and Mental Health of Healthcare Workers. International journal of environmental research and public health, 21(11), 1533. https://doi.org/10.3390/ijerph21111533	A cross-sectional study of 206 doctors and nurses at Adıyaman Training and Research Hospital who provided emergency services in the aftermath of the February 2023 Turkey-Syria earthquakes	The study examined PTSD, sleep, depression, and anxiety in physicians and nurses in Adıyaman, one of the worst affected cities	Burnout not explicitly assessed	Vulnerability of doctors and nurses was assessed with PTSD Short Scale, Beck Anxiety Inventory (BAI), Beck Depression Inventory (BDI), and the Pittsburgh Sleep Quality Index (PSQI. All scores were higher in females and in those personally impacted by earthquake. BAI, PTSD and PSQI scores of nurses were higher than those of doctors	Suggested care models to support long-term rehabilitation and monitoring programs. These should incorporate stress management and resilience training into disaster preparedness programs to enhance healthcare workers’ coping skills for traumatic events	Protect the mental health of doctors and nurses after earthquakes to sustain effective healthcare services Governments should take greater responsibility for supporting the mental health of healthcare workers to prevent disaster burnout
14	Akdağ, B., Bozduman Çelebi, S., & Kılıçaslan, F. (2024). Investigation of Post-Traumatic Stress Disorder, Secondary Traumatic Stress and Burnout in Child/Adolescent Psychiatrists After the Kahramanmaraş Earthquake. Dicle Medical Journal, 51(4), 565–572. https://doi.org/10.5798/dicletip.1608133	Cross-sectional quantitative. 41 child/adolescent psychiatrists working in disaster areas (July–Sept 2024)	To assess PTSD, secondary traumatic stress, and burnout in child/adolescent psychiatrists post-earthquake	Copenhagen Burnout Inventory (CBI) used. 48.8% showed moderate–high burnout	PTSD Short Form and Secondary Traumatic Stress Scale used. High scores linked to personal loss or damage	No PEI model defined. Relationship between personal trauma and burnout discussed	Recommends trauma-focused therapies, supervision, peer support, and longitudinal research
15	Topkara, F. N., Aktaş-Reyhan, F., Dağlı, E., & Bakır, E. (2024). Determining the Relationship Between Compassion Fatigue and Secondary Traumatic Stress Levels Among Healthcare Workers Serving in Earthquake Zones TOGU Health Sciences Journal, 4(2), 152–165 10.52369/togusagbilderg.1418440	Descriptive cross-sectional study. 475 healthcare workers (midwives, nurses, doctors) in Hatay (May–Aug 2023)	To examine the relationship between compassion fatigue and secondary traumatic stress	Compassion Fatigue Short Scale used. Mean score: 71.7 ± 28.3	Secondary Traumatic Stress Scale used. Mean score: 55.6 ± 12.3. Higher in those with damage/loss	No defined PEI model. No structured prevention/intervention framework	Recommends psychosocial support, financial incentives, and enhanced social backing
16	Selvi, Ö., & Aslan, A. (2024). Evaluation Of the Relationship Between Post-Traumatic Stress and Psychological Resilience of Healthcare Workers Who Served During the February 6, 2023 Maraş Earthquake. Middle Black Sea Journal of Communication Studies, 9(1), 1–18. https://doi.org/10.56202/mbsjcs.1488476	Cross-sectional quantitative. 200 healthcare workers (doctors, nurses, paramedics) from Kırıkkale deployed to earthquake zone (sample: 140)	To examine the relationship between PTSD and resilience	Burnout not directly assessed. PTSD measured using the Post-Traumatic Stress Diagnostic Scale (mean: 17.55 ± 12.33)	Psychological Resilience Scale for Adults used (mean: 122.03 ± 15.27). Vulnerability linked to mental health history and gender	No explicit PEI model. Suggests mental health screening and resilience-based planning	Recommends psychosocial interventions, gender-sensitive planning, and screening before deployment
17	Urşan, G., Çiçekoğlu Öztürk, P., & Büyükbayram Arslan, A. (2024). The Effect of Secondary Traumatic Stress and Burnout Levels on the Psychological Resilience of Emergency Department Workers. *Journal of Samsun Health Sciences, 9*(3), 333–353. https://doi.org/10.47115/jshs.1524499	Cross-sectional study conducted in two emergency departments in Turkey (May–Oct 2023). N = 80 doctors and nurses	To examine the effect of burnout and secondary traumatic stress on resilience	Assessed using Maslach Burnout Inventory (MBI). Mean score: 47.25 ± 11.09	Secondary Traumatic Stress Scale (STSS) and Psychological Resilience Scale for Adults used. Mean STSS: 32.93 ± 11.73; Resilience: 137.81 ± 15.67. 32.5% had served in the earthquake zone	No formal PEI model defined but recommends early identification of trauma symptoms and resilience-based training	Proposes in-service training, trauma-awareness programs, and supportive work environments for emergency staff
18	Karaca, T., & Aydın Özkan, S. (2024). Moral sensitivity, spiritual care perception, and compassion fatigue of nurses caring for earthquake victims. *International Nursing Review*. https://doi.org/10.1111/inr.13066	Descriptive-correlational design. N = 483 hospital-based nurses in 10 Turkish provinces impacted by the 2023 earthquake (April–Sept 2023)	To explore nurses’ compassion fatigue in relationship with their perception of spiritual care and moral sensitivity,	Compassion Fatigue Short Scale (CFSS) used. Mean CFSS score: 88.08 ± 2.76; burnout subscore: 53.37 ± 3.37	Personal vulnerability of nurses as survivors was not explored in this study	No PEI model has been explicitly identified	Calls for seminars, in-service training, and institutional strategies to reduce compassion fatigue. Nurses also need specific policies to monitor their resilience and to support self‐care

#### Interrater reliability

Consistent with the approach described by Tricco et al. (2016), we assessed the interrater reliability as a measure of validity. The Cohen’s kappa score for reviewer agreement during the title and abstract screening stage was 0.80.

### Step 4: charting the findings

Guided by the review question, our team discussion identified the variables for data extraction. Afterwards, an analytical framework for data charting was produced by pooling and then piloting the preliminary results. The 18 included articles were charted in Microsoft Excel for Mac 2021 version 16.53, using the following three column headings: 1) “Authors' information” (full APA citation); 2) “Study Design & Participants”; and 3) “Research Aims”. After further analytical discussion, four more columns (4, 5, 6, and 7) were created and added to the extraction template (see Stage 5). All charting in columns 1–3 was completed independently by two authors (UBK and EO). The charted data are displayed in Table 2.

### Step 5: collating, summarizing, and reporting the results

Following the Levac et al. (2010) recommendations. We used thematic analysis to identify patterns within our data set; coding for analytical themes that related results to the research question and summarizing research, policy, and practice implications for future research. The team held regular interdisciplinary triangulation meetings, i.e. authors from different disciplines held weekly meetings to discuss possible meanings, and interpretations, generating new insights from data. The objective of these meeting was to create a coherent framework for interdisciplinary evaluation, to explore the convergence of perspectives from researchers in various fields, to analyze data, to avoid monodisciplinary bias and limitations (Haeussler and Sauermann, 2020) and to broaden the conceptualization and interpretation of the constructed themes (Patton, 1999; Tiainen and Koivunen, 2006). We identified four emerging themes that, to a large extent, align with our review question: a) Burnout of doctors and nurses; b) Vulnerability of doctors and nurses as survivors; c) PEI Models used, evaluated, or suggested; and d) Research, practice, and/or policy recommendations. Each theme included three operationalized codes or indicators. If needed, we can provide in a in the Supplementary Material a table with our codes or indicators. We used the four themes as headings for Table 2 Columns 4, 5, 6, and 7. After separately extracting the data for the columns, six authors (EOC; MK; BC; FR; BBB; and PMK) compared them to ensure accuracy and consistency.

## Results

Our search yielded 104 unique records. Full-text documents were examined for 32 of these, and ultimately 18 articles were included for analysis (Fig. 1). Of the included studies, 1 was a qualitative survey, 6 were semi structured interviews, 7 cross-sectional studies (3 quantitative; and 4 descriptive), 1 descriptive-correlational study, 1 scoping review, 1 multicenter study, and 1 performance evaluation study. Characteristics of the included studies can be seen in Table 2. Years of publication ranged from 2003 to 2024. Across the 18 studies reviewed, the aims were largely centered on documenting the psychological and occupational effects of earthquakes on doctors and nurses, with several focusing on resilience, secondary traumatic stress, and burnout as measurable outcomes. 3 quantitative studies assessed the prevalence of post-traumatic stress disorder, depression, anxiety, compassion fatigue, and sleep disturbances among healthcare professionals working either directly in the disaster zone or in referral hospitals, often using standardized instruments such as the Maslach Burnout Inventory (MBI), the Copenhagen Burnout Inventory (CBI), Turkish version of the Nurse Stress Scale (NSS), Perceived Ability to Cope with Trauma (PACT) Scale, Secondary Traumatic Stress Scale (STSS), Brief Psychological Resilience Scale (BRS), Depression and Anxiety Stress Scale–21 (DASS21), or PTSD scales. Complementing these, 11 qualitative investigations explored the lived experiences of nurses and physicians before, during, and after their deployment in disaster zones, highlighting issues such as inadequate preparedness, role ambiguity, resource shortages, and the emotional burden of witnessing mass casualties. The scoping review broadened this picture by identifying the particular vulnerability of healthcare workers as a subgroup within larger populations affected by the earthquakes, while technology-focused research introduced digital and GIS-supported tools as potential means of improving coordination and communication in emergency response.

**Fig. 2:**
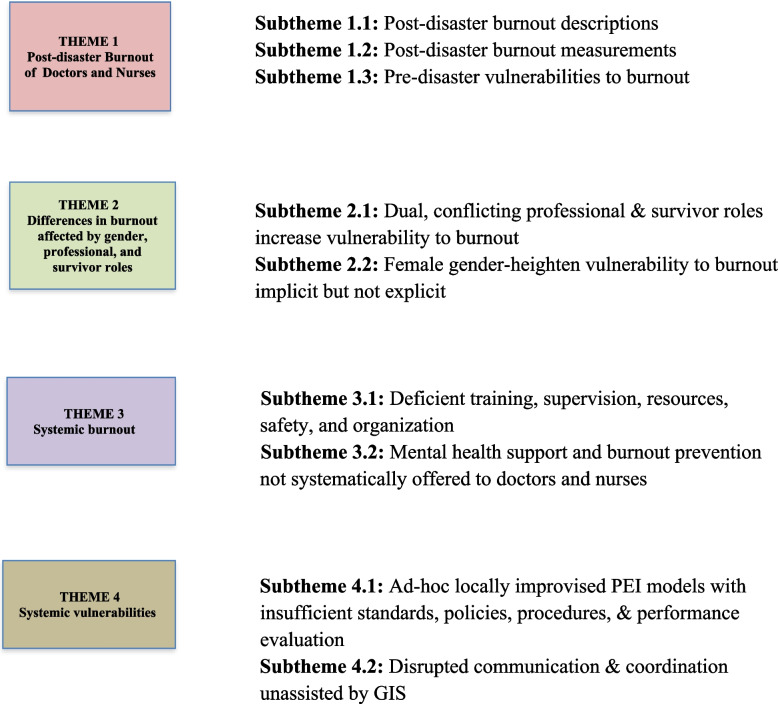
Themes and subthemes

Together, these study aims illustrate a diverse but overlapping research agenda that has sought to capture both the individual-level psychological toll and the system-level gaps in preparedness and response across the Turkiye earthquake context. Collectively, these 18 studies contribute to the development of a comprehensive framework for understanding the past, present, and future impacts of disasters on healthcare professionals. This framework organizes the reviewed literature into four thematic domains: (1) post-disaster burnout of doctors and nurses, (2) differences in burnout affected by gender, professional, and survivor roles, (3) systemic burnout, and (4) systemic vulnerabilities (as seen in Fig. 2). The subthemes are detailed in the Discussion section.

## Discussion

### Theme 1: post-disaster burnout of doctors and nurses

Both quantitative and qualitative studies document the overwhelming toll on doctors and nurses. Sert et al. (2023) and Çetinkaya Özdemir et al. (2023) found that exhaustion was a significant problem for nurses during disaster response. Akçay et al. (2025) reveal that more than half of the participants experienced burnout and distress in the early aftermath of the disaster, a figure echoed by Satılmış, D., Yıldız, E., & Cevik, E. (2024), who identified high rates of PTSD among frontline professionals. Qualitative narratives add another layer of detail, highlight how, in the overwhelming context of destruction and loss, the fear, anger, and helplessness of providers translated directly into burnout with a substantial drop in their professional performance with varying degrees of compassion fatigue and resilience [49].

#### Subtheme 1.2: post-disaster burnout measurements

While not consistently assessed across all reviewed studies, burnout evidence points to high emotional exhaustion, stress, and depression, especially among nurses, due to inadequate disaster training, unsafe clinics, insufficient resources, and chaotic coordination. Using tools such as the Maslach Burnout Inventory (MBI), the Depression, Anxiety, and Stress Scale–21, and the Brief Resilience Scale in some studies found that over half of participants experienced high levels of burnout and low resilience [50, 51]. Overall, the lack of preparedness, poor crisis management, and environmental hardships significantly contributed to psychological distress and burnout among disaster healthcare workers. In this review, nurses had higher emotional exhaustion, depression, and stress scores than doctors, as well as lower self-reported resilience. Among doctors, child and adolescent psychiatrists have shown high burnout rates in the Copenhagen Burnout Inventory [52]. Using the Compassion Fatigue Short Scale (CFSS) to explore the relationships between moral sensitivity, compassion fatigue, and burnout among nurses caring for earthquake victims, Karaca, T., & Aydın Özkan, S. (2024), as well as Topokara et al. (2024), also point out high burnout in nurses.

#### Subtheme 1.3: pre-disaster vulnerabilities to burnout

Some studies point to vulnerabilities that predate the earthquake itself, particularly in relation to gender, resilience, and existing psychological strain. Özbay, A., & Bülbül, A. E. (2025) demonstrate how resilience and gender can mediate the stress response of mental health professionals, suggesting that the workforce carries uneven protective resources even before deployment, while Selvi, Ö., & Aslan, A. (2024) and Urşan et al. (2024) show that secondary traumatic stress and burnout can erode resilience, a finding amply shown in other studies [53]. Sani Mert, I., & Koksal, K. (2025) identify five recurring themes that predispose nurses to burnout: moral obligation, driving them to prioritize others at the expense of their own wellbeing; a high but unsustained motivation due to cumulative strain; insufficient experience, which undermines their confidence in disaster settings; balancing responsibilities, where family duties and professional expectations collide; and preparation challenges, where limited training and resources leave them vulnerable to stress escalation. Together, these findings suggest that pre-disaster vulnerabilities are evident, structural realities, making burnout not only a post-disaster outcome, but a foreseeable consequence that pre-exists a crisis.

### Theme 2: differences in individual burnout affected by gender, job, and survivor roles

#### Subtheme 2.1: dual, conflicting nurse and survivor roles increase vulnerability to burnout

In our study, Sani Mert, I., & Koksal, K. (2025) and Emirza et al. (2024) emphasize that nurses not only carried the burden of their professional roles but also suffered from their own trauma as individual survivors, or as members of families who experienced losses or live victims, creating what they describe as “secondary traumatization.” Özbek Güven et al. (2024) show that these experiences of chain reaction traumas influenced even fundamental beliefs of dignified care and a good death, while Karaca, T., & Aydın Özkan (2024) link compassion fatigue and moral injury to the psychosocial exhaustion of nurses. These findings suggest that the disaster environment introduces dual strain: the external pressure of resource scarcity and overwhelming demand, and the internal conflict of being both a survivor and caregiver, resulting in a rapid escalation of burnout. While nurses and doctors both face elevated risks, research indicates that female nurses may experience a disproportionately higher burden in post-disaster settings [54] and as such, nurses appear to be more vulnerable to burnout than physicians. Lee & Friese (2021) documents that compared with female physicians, female nurses are at a 70% higher risk of suicide in the United States.

#### Subtheme 2.2: female gender-heighten vulnerability to burnout implicit but not explicit

It was surprising to see that of the eighteen reviewed articles, not a single one has noted that gender was a significant modulator of vulnerability to burnout. While most had abundantly shown that nurses (of which the majority have been females across all studies) had the added burden of caring for their parents, husbands, and children while also caring for disaster patients, not a single sentence explicitly pointed out that female gender carried added burnout risks. Future research should examine whether cultural norms may influence how gendered vulnerabilities are reported. This also affects and suggests that Turkish researchers may have felt the unnecessary need to emphasize that traditional Turkish culture assumes the women’s responsibility of caring for family members. This gender lapse stands out in contradiction to past and current literature. Mussida, C., & Patimo, R. (2021) point out to a disproportionate gender responsibility in disasters [55]. Women are the primary caregivers for children and elderly parents; in post-disaster these domestic responsibilities intensify alongside increasing job demands, creating severe challenges to balance personal life priorities with disaster work. The research of Lyubarova et al. (2023) indicates that women have less control over their workloads and schedules compared to men, and that this lack of autonomy, a key driver of burnout, is more commonly reported by female doctors and nurses [56]. Other researchers report a higher emotional investment and higher levels of emotional exhaustion, as women are more likely to explore the psychosocial issues of patients. These additional levels of drain, lead to higher rates of compassion fatigue and burnout [57]. Female physicians are more likely than male physicians to experience depression, burnout and suicidal ideation [58]. Ultimately, many studies show that female physicians consistently feel less valued by their organizations than their male counterparts, a factor strongly linked to burnout [59–62]. Irrespective of profession, nurses or physicians, the burnout risk is clearly higher in female professionals.

### Theme 3: systemic burnout

#### Subtheme 3.1: deficient training, supervision, resources, safety, and organization

When an earthquake hits, pre-existing vulnerabilities in local hospitals or field mobile clinics are abruptly amplified. Despite recognition for their rapid responses, excellent communication skills, and innovative problem-solving in disaster zones [16], persistent gaps in nursing preparedness training do exist [63, 64]. As reflected in our review by Deniz Doğan et al. (2024), nurses faced challenges of inadequate organization, lack of resources, and work beyond hospital capacity, often accompanied by uncertainty in adapting to disaster-specific care. Some common themes were insufficient training to cope with anxiety and stress, uncertainty concerning treatment, lack of staff support, and inadequate skills to address the emotional struggles of patients and their families. These findings echo those of other studies, which found gaps in disaster core competencies among Turkish nurses [65], indicating a need for improved disaster preparedness [66].

#### Subtheme 3.2: mental health support and burnout prevention not systematically offered to doctors and nurses

Our review has abundantly found that nurses experienced high anxiety and sadness while carrying extreme workloads under adverse conditions like unsafe hospital buildings and staff shelters, poor hygiene, cold weather, disrupted communication and chaotic coordination; aside from prayer and talking to family, most did neither knew how to manage their anxiety, nor did they receive any mental health support [67]. In the absence of psychological support, the prolonged emotional and mental exhaustion eroded empathy for patients [68]. The findings are consistent with those of other studies, pointing out that prolonged and overwhelming suffering can lead to compassion fatigue [69, 70]. The intense time pressure of triage and the different approaches to patient care can lead to high-stress disagreements between doctors and nurses [71]. Inadequate communication of critical patient information, due to verbal orders without paper records [72], leads to fragmented care and potential errors, fueling tension [73]. As noted in our review, this can lead to a breakdown in trust and conflict between nurses and physicians. In the absence of skilled psychological support, this can further increase burnout among doctors [74] and nurses [75, 76].

### Theme 4: systemic vulnerabilities

#### Subtheme 4.1: ad-hoc locally improvised local PEI models with insufficient standards, policies, procedures, performance evaluation

A recurring finding across the studies reviewed that while the psychological and occupational burden of earthquakes on healthcare professionals has been consistently documented, the PEI models intended to prevent or mitigate these effects remain fragmented, inconsistently applied, and rarely evaluated. Balikuddembe et al. (2024) point to the absence of streamlined frameworks addressing the specific vulnerabilities of healthcare workers, noting that post-disaster interventions were often locally improvised rather than guided by evidence-based established models [77]. Satılmış et al. (2024), emphasize that even though Turkey’s national disaster response plan officially mobilized, mental health and burnout prevention for professionals were not systematically integrated, leaving doctors and nurses without adequate support [78]. Yanik & Ediz (2024) add that volunteer nurses, while crucial in the disaster response, remained without clear training modules, coordinated supervision, or defined guidelines, resulting in uneven preparedness and heightened distress. Çifçi & Kilinç, 2024 reinforce this by underlining the government’s responsibility to develop sustainable care models that include long-term monitoring and rehabilitation, while Akdağ et al. (2024) and Topkara et al. (2024) argue that interventions should also be longitudinal, trauma-focused, and inclusive of peer-support structures [79]. The repeated references to training deficits, lack of supervision, and weak institutional backing across qualitative accounts such as Düzgün et al. (2023), Deniz Doğan et al. (2024), and Emirza et al. (2024) further show that prevention efforts remain individualized and local, rather than systemic.

#### Subtheme 4.2: uncertain, disrupted communication & coordination unassisted by GIS

In disasters, the speed of action exceeds the flow of supporting information [80] and therefore recovery processes are expedited and ultimately reduced. In our review, poor communication was emphasized as a major deficiency in coordination of disaster response with one study stating, “…communication is vital. It was a tragedy that those who could not even combine two words were trying to coordinate” Another participant described their experience, saying, “A vast uncertainty. Lack of coordination. Not knowing what is lacking, where, and by who is terrifying" [68]. This resonates with the accounts of Deniz Doğan et al. (2024), Düzgün et al. (2023), and Emirza et al. (2024), who all describe confusion, exhaustion, and moral distress caused by inadequate organization, inconsistent communication, and lack of structured preparedness [49, 81, 82]. Akçay et al. (2025) and Yanik & Ediz (2024), further add that disparities in training and the absence of unified protocols across teams created fragmented responses, leaving nurses and doctors with a heavier workload and higher burnout [50, 83]. Although most studies pointed to an overwhelming organizational breakdown, very few proposed technological solutions. The exception is provided by [84], who evaluated the HERA platform, a GIS-supported disaster health information system capable of monitoring the real-time status of 537 healthcare facilities and enabling rapid communication through digital channels. Their findings indicate that by reducing uncertainty, allowing quicker referrals, and supporting coordinated task allocation, such a system can indirectly reduce the psychological burden on professionals who would otherwise struggle with a chaotic information flow.

## Limitations

Our scoping review was limited to studies published in peer-reviewed journals in Türkiye and abroad, written in English or Turkish. While our study reviewed many qualitative descriptions of individual professionals’ experience, it lacked the benefit of established PEI, which tend to be detailed, longitudinal, and highly contextual. We may also have missed PEIs that were used locally but not published outside “grey literature” (e.g., institutional reports at city or provincial levels). Data extraction was based on information reported in articles, so variables that were inadequately or inconsistently reported may have led to incomplete classification. Despite these limitations, this scoping review offers new insights into knowledge production on post-disaster burnout among Turkish doctors, nurses, and health systems.

## Conclusions

In this study, we developed a grasp of the complex interplay of personal, professional, and systemic factors contributing to doctors' and nurses' vulnerability to post-earthquake burnout in Türkiye over the past three decades. We identified significant gaps in preventive and early interventions. Most of the PEI programs under review focused on situation evaluation, while the development of individual skills; monitoring and evaluating PEI performance, PEI enhancements, and evidence-based policies were seldom included. This scoping review underscores the substantial psychological burden experienced by healthcare professionals following earthquakes, with consistent evidence of elevated burnout, emotional exhaustion, and secondary traumatic stress—particularly among nurses. While the prevalence and contributing factors of burnout are well documented, our review highlights a critical gap between the documented burden of burnout and the very limited development of structured PEI models, particularly those incorporating GIS or other system-level tools The structural and functional diversity of PEI in our study has limited knowledge transfer and scalability in field applications, making comparative synthesis challenging due to the absence of a common nomenclature and established measurement instruments. In disaster settings, the challenges of infodemic, misinformation, and the uncontrolled spread of unverified content through social media can be effectively addressed only with reliable geographic information systems (GIS). As GIS-supported methodologies have frequently been overlooked, timely and realistic dynamic risk mapping to inform subsequent decisions was absent in the studies reviewed. This study underscores the need for a multilayered GIS that enables healthcare professionals and policymakers to access data on both Turkish citizens and refugees living in the affected areas.

Remarkably, few studies move beyond descriptive analysis to propose or assess system-level strategies aimed at mitigating these outcomes. In particular, the near absence of approaches incorporating GIS or other data-driven tools highlights an important missed opportunity in disaster response planning. Such tools may offer potential for identifying high-risk populations, allocating resources more effectively, and supporting timely interventions.

These findings point to a disconnect between the recognition of burnout as a significant public health concern and the limited advancement of coordinated, evidence-based responses. Future research should prioritize the design, implementation, and evaluation of structured PEI frameworks, including the potential integration of system-level and geospatial approaches, to better support healthcare workers in disaster-affected settings.

Making definitive recommendations is beyond the scope of work of this scoping review. The gaps the review has identified can only suggest directions for future research. As such, creating quickly adaptable, empirically supported, and technologically enabled PEI models would lessen the psychological toll that earthquakes take on medical personnel. Earthquake-prone and vulnerable. Türkiye could benefit from prioritizing gender-sensitive, socio-ecological designs, multidisciplinary cooperation, community stakeholder participation, and GIS-based monitoring of burnout and resilience indicators. The creation of a nationwide network of burnout prevention centers could serve as a resource and guide for clinical and policy decision-making by enhancing stakeholder-informed communication and coordination. It would also foster a much-needed sense of security for vulnerable communities and healthcare professionals.

## Data Availability

No data was generated for this study. All data analyzed for the purpose of this study are included in this published article (Table 2).
